# Highly efficient L-lactate production using engineered *Escherichia coli* with dissimilar temperature optima for L-lactate formation and cell growth

**DOI:** 10.1186/1475-2859-13-78

**Published:** 2014-05-29

**Authors:** Dandan Niu, Kangming Tian, Bernard A Prior, Min Wang, Zhengxiang Wang, Fuping Lu, Suren Singh

**Affiliations:** 1 Key Laboratory of Industrial Fermentation Microbiology, Ministry of Education & The College of Biotechnology, Tianjin University of Science & Technology, Tianjin 300457, China; 2 Department of Microbiology, Stellenbosch University, Private Bag X1, Matieland 7602, South Africa; 3 Department of Biotechnology & Food Technology, Faculty of Applied Sciences, Durban University of Technology, P.O. Box 1334, Durban 4001, South Africa

**Keywords:** L-Lactate, L-Lactate dehydrogenase, Enzymatic thermodynamics, Metabolic engineering

## Abstract

L-Lactic acid, one of the most important chiral molecules and organic acids, is produced via pyruvate from carbohydrates in diverse microorganisms catalyzed by an NAD^+^-dependent L-lactate dehydrogenase. Naturally, *Escherichia coli* does not produce L-lactate in noticeable amounts, but can catabolize it via a dehydrogenation reaction mediated by an FMN-dependent L-lactate dehydrogenase. In aims to make the *E. coli* strain to produce L-lactate, three L-lactate dehydrogenase genes from different bacteria were cloned and expressed. The L-lactate producing strains, 090B1 (B0013-070, Δ*ldhA*::*diflldD*::P_*ldh*_-*ldh*_Lca_), 090B2 (B0013-070, Δ*ldhA*::*diflldD*::P_*ldh*_-*ldh*_Strb_) and 090B3 (B0013-070, Δ*ldhA*::*diflldD*::P_*ldh*_-*ldh*_Bcoa_) were developed from a previously developed D-lactate over-producing strain, *E. coli* strain B0013-070 (*ack-ptappspflBdldpoxBadhEfrdA*) by: (1) deleting *ldhA* to block D-lactate formation, (2) deleting *lldD* to block the conversion of L-lactate to pyruvate, and (3) expressing an L-lactate dehydrogenase (L-LDH) to convert pyruvate to L-lactate under the control of the *ldhA* promoter. Fermentation tests were carried out in a shaking flask and in a 25-l bioreactor. Strains 090B1, 090B2 or 090B3 were shown to metabolize glucose to L-lactate instead of D-lactate. However, L-lactate yield and cell growth rates were significantly different among the metabolically engineered strains which can be attributed to a variation between temperature optimum for cell growth and temperature optimum for enzymatic activity of individual L-LDH. In a temperature-shifting fermentation process (cells grown at 37°C and L-lactate formed at 42°C), *E. coli* 090B3 was able to produce 142.2 g/l of L-lactate with no more than 1.2 g/l of by-products (mainly acetate, pyruvate and succinate) accumulated. In conclusion, the production of lactate by *E. coli* is limited by the competition relationship between cell growth and lactate synthesis. Enzymatic properties, especially the thermodynamics of an L-LDH can be effectively used as a factor to regulate a metabolic pathway and its metabolic flux for efficient L-lactate production.

**Highlights:**

The enzymatic thermodynamics was used as a tool for metabolic regulation. ► minimizing the activity of L-lactate dehydrogenase in growth phase improved biomass accumulation. ► maximizing the activity of L-lactate dehydrogenase improved lactate productivity in production phase.

## Introduction

L-Lactic acid, one of the two lactic acid optical isomers, is produced via pyruvate from carbohydrates in diverse microorganisms catalyzed by an NAD^+^-dependent L-lactate dehydrogenase [1]. L-Lactic acid is generally supplemented to foods or feeds as an excellent sour agent or pH modulator [2]. L-Lactic acid is also an important precursor for synthesis of chiral compounds such as chiral drugs and chiral pesticides [3,4] and, more importantly, as a monomer for the synthesis of poly-L-lactic acid, a bio-degradable and environmental friendly polymer [2,5].

In contrast to past applications of racemic lactic acid, L-lactic acid as a monomer for poly-L-lactic acid synthesis must possess the highest optical purity and chemical purity [6,7], Recently L-lactate production by microbial has been extensively investigated for this reason.

In previous works, metabolically engineered *E. coli* has been shown to be a suitable host for large-scale production of D-lactic acid or L-lactic acid, and several metabolically-engineered *E. coli* strains have been successfully constructed for efficient synthesis of D-lactic acid or L-lactic acid of high optical [8-12]. For L-lactic acid formation, *E. coli* strains are usually genetically modified to: (1) create a pathway for L-lactic acid formation, (2) block the pathway for L-lactic acid catabolism, and (3) construct/block pathways connected with intermediates for L-lactic acid [12-16].

Generally, L-lactate (and some other organic acids) is formed during cell growth, which negatively affects cell activity and cell growth and, as a consequence, exerts a detrimental effect on L-lactate titer and yield. The L-lactic acid synthesis pathway being less active during cell growth could be positive for cell growth and finally for L-lactic acid titer and yield as this strategy has been confirmed to be effective in D-lactate synthesis in *E. coli *[11,17,18].

It is expected that effectively controlling intracellular lactate dehydrogenase activity is crucial to achieve efficient lactate synthesis under fermentation process. Here, we described a new strategy to assess this proposal by engineering an L-lactate synthesis pathway in *E. coli* with temperature optima different between cell growth and a heterologously expressed bacterial L-lactate dehydrogenase. The strain expressed an L-lactate dehydrogenase from a thermophilic bacterium showed robust growth at its temperature optimum and was more efficient in fermenting glucose to L-lactate with less by-products formation at an elevated temperature.

## Materials and methods

### Strains

The genotypes of the microbial strains and plasmids used in the present study are summarized in Table 1. *Escherichia coli* strain 070 (Δ*ack-pta*::*dif*Δ*pps*::*dif*Δ*pflB*::*dif*Δ*dld*::*dif*Δ*poxB*::*FRT*Δ*adhE*::*dif*Δ*frdA*::*dif*) was reported previously [10]. Strain 090B1 (070, Δ*ldhA*::*diflldD*::*ldhAp-ldh*_Lca_), 090B2(070, Δ*ldhA*::*diflldD*::*ldhAp-ldh*_Strb_) and 090B3(070, Δ*ldhA*::*diflldD*::*ldhA*p-*ldh*_Bcoa_) were constructed during this study. DNA manipulations were performed using conventional techniques [19].

**Table 1:** Strains and plasmids used in this study

**Strains**	**Relevant characteristics**	**Source or reference**
*Escherichia coli* B0013-070	B0013, Δ*ack-pta*, Δ*pps*, Δ*pflB*, Δ*dld*, Δ*poxB*, Δ*adhE*, Δ*frdA*	[10]
*E. coli* B0013-080C	B0013-070, Δ*ldhA*	This study
*E. coli* B0013-090B1	B0013-080C, Δ*lldD*:: *ldhA*p-*ldh*_Lca_	This study
*E. coli* B0013-090B2	B0013-080C, Δ*lldD*:: *ldhA*p-*ldh*_Strb_	This study
*E. coli* B0013-090B3	B0013-080C, Δ*lldD*:: *ldhA*p-*ldh*_Bcoa_	This study
*Bacillus coagulans* B1821	Wild type	CICIM-CU^a^
*Lactobacillus casei* B1192	Wild type	CICIM-CU^a^
*Steptococcus. bovis* 1.1624	Wild type	CGMCC^b^
*Plasmids*	*Relevant characteristics*	*Source or reference*
pSK-*dif*Gm^R^	*dif*Gm, *bla*	[20]
pMD18-T simple	*bla*; TA cloning vector	TaKaRa, Japan
pLDHex	*bla*; P_*ldhA*_, T_*ldhA*_	This study

^a^The Culture and Information Center of Industrial Microorganisms of China Universities, Jiangnan University, China.

^b^China General Microbiological Culture Collection Center, Institute of Microbiology, CAS, Beijing, China.

To construct the 090B1, 090B2 and 090B3, *ldhA* in strain 070 encoding a D-lactate dehydrogenase for conversion of pyruvate to D-lactate was deleted followed by placing *ldhAp-ldh*_Lca*,*_*ldhAp-ldh*_Strb_ or *ldhA*p-*ldh*_Bcoa_ expression cassette which encodes an L-lactate dehydrogenase under the control of *ldhA* promoter for conversion of pyruvate to L-lactate from different microorganisms in the middle of the chromosomal *lldD* gene in strain 080C (070, Δ*ldhA*::*dif*).

### Deletion of *ldhA* to obtain *E. coli* B0013-080C

The *ldhA'* gene was cloned from the genomic DNA of *E. coli* B0013-070 using PCR amplification and the primers LdhA1 and LdhA2. The PCR product was purified and cloned into pMD18-T simple vector to yield plasmid pMD-ldhA'. This plasmid was digested with *Sal*I and *Kpn*I and blunted by incubation with *Pfu* DNA polymerase, which was then ligated with a selectable marker (Gm^R^ with *dif* sites flanked) isolated from pSK-*dif*Gm^R^ [10], to yield a hybrid plasmid pMD-ldhA::Gm, in which a 212 bp fragment in the middle of *ldhA* in pMD-ldhA was removed and replaced with *dif*Gm. The deletion cassette, *ldhA*'-*dif*Gm-*dif*-*ldhA'*, was recovered from pMD-ldhA::Gm with *Eco*RI digestion and agarose gel isolation. The deletion cassette was electro-transformed into strain B0013-070 and *ldhA* disruption mutant was selected by the method described previously [10,11]. The resulting recombinant strain was designated 080C (B0013-070, Δ*ldhA*::*dif*).

### Expression of *ldh* and disruption of *lldD*

The *E. coli ldhA* gene was cloned from the genomic DNA of B0013-070 using PCR amplification and the primers LdhA3 and LdhA4. The resulting 1.6-kb PCR fragment, which included the promoter, the structural region of the *ldhA* gene and the terminator, was inserted into the pMD18-T simple vector to create pMD-ldhA. The reverse PCR fragment from plasmid pMD-ldhA was amplified using the primers RldhA1 and RldhA2. The amplified fragment was then self-ligated to create an expression plasmid, pLDHex. The *ldh*_Bcoa_ gene encoding a *Bacillus coagulans* L-LDH was recovered from the genome by PCR amplification with primers BcoaLDH1 and BcoaLDH4. After digestion with *Bam*HI and *Eco*RI, this 885-bp fragment was cloned into the *Bgl*II and *Eco*RI sites of pLDHex to create pLDH-ldh_Bcoa_. A selectable marker, *dif*Gm (Gm^R^ with *dif* sites flanked) isolated from pSK-*dif*Gm^R^ [10], was subcloned into the *Eco*RV site of pLDH-ldh_Bcoa_ to create pLDH-ldh_Bcoa_-Gm. Similarly, pLDH-ldh_Strb_-Gm was created by cloning a 1,464-bp *ldh*_Strb_ (encoding a *Streptococcus bovis* L-LDH) digested with *Bam*HI and *Eco*RI into the *Bgl*II and *Eco*RI sites of pLDHex followed by inserting *dif*Gm into the *Eco*RV site. For development of pLDH-ldh_Lca_-Gm, PCR amplified 1.26-kb fragment of *ldh*_lca_ (encoding a *Lactobacillus casei* L-LDH) was first cloned into the *Bgl*II and *Pst*I sites of pLDHex to yield pLDH-ldhLca followed by insertion of *dif*Gm from pSK-*dif*Gm^R^ [10] into the *Eco*RV site. Meanwhile, the whole length of *lldD* was amplified from the genomic DNA of *E. coli* B0013 with primers LldD1 and LldD2. The resulting PCR 1154-bp fragment was cloned into pMD18-T simple vector to create pMD-*lldD*. Afterwards, the *ldh* expression cassette (P_*ldhA*_-*ldh*-Gm) was isolated from pLDH-ldh_Lca_-Gm, pLDH-ldh_Strb_-Gm, or pLDH-ldh_Bcoa_-Gm with *Bam*HI digestion and gel purification and cloned into the *Bam*HI sites of pMD-lldD to create pMD-lldD::P_*ldhA*_-*ldh*_Lca_-Gm, pMD-lldD::P_*ldhA*_-*ldh*_Strb_-Gm, or pMD-lldD::P_*ldhA*_-*ldh*_Bcoa_-Gm, in which a 39-bp *Bam*HI-fragment was deleted in the middle of *lldD*. These plasmids were digested with *Sma*I and the resulting deletion/expression cassettes were gel isolated and electroporated into strain B0013-080C The recombinants were selected by the method described previously [10,18]. The resulting recombinant strains were designated 090B1 (B0013-080C, Δ*lldD*::P_*ldhA*_*-ldh*_Lca_), 090B2 (B0013-080C, Δ*lldD*::P_*ldhA*_*-ldh*_Strb_), and 090B3 (B0013-080C, Δ*lldD*::P_*ldhA*_*-ldh*_Bcoa_).

Details of the primers used in this study are provided in Table 2.

**Table 2:** Primers used in this study

**Primers**	**Sequence (5'-3') restriction sites are italic/underlined**	**Restriction sites**
LdhA1	TAA*GAATTC*^a^TTATGAAACTCGCCGTTTATAGCACA	*EcoR*I^a^
LdhA2	CTT*GAATTC*^a^*AAGCTT*^a^GCTGCCGGAAATCATCATTTTTT	*EcoR*I^a^, *Hin*dIII^a^
LdhA3	GGGCAGCCCGAGCGTCATCAG	
LdhA4	GCTGCCGGAAATCATCATTTTTT	
BcoaLDH1	CC*GGATCC*^a^AATCAGGGTGTTGCAGAAGAGCTTG	*Bam*HI^a^
BcoaLDH4	GCG*GAATTC*^a^TTACAATACAGGTGCCATCGTTTCT	*EcoR*I^a^
LcaLDH1	CGC*GGATCC*^a^AGTATTACGGATAAGGATCAC	*Bam*HI^a^
LcaLDH4	CGC*CTGCAG*^a^TCCTGTTCTTCGTTTG	*PstI*^a^
StrbLDH1	CGC*GGATCC*^a^ACTAAACAACACAAAAAAG	*Bam*HI^a^
StrbLDH2	CCG*GAATTC*^a^TACAGGGATTGTTGCCGCA	*EcoR*I^a^
LldD1	GG*CCCGGG*^a^CATGATTATTTCCGCAGCCA	*Sma*I^a^
LldD2	GG*CCCGGG*^a^CAGGCAACTCTTTACCCAGCCC	*Sma*I^a^

^a^Restriction sites with corresponding restriction enzyme.

### Measurement of LDH activity

Strains (stored as glycerol stocks at −80°C) were first grown on Luria-Bertani (LB) plates for approximately 24 h at 37°C and then the colonies were transferred to 50 ml of LB medium in a 250-ml flask. After growing while shaking at 200 rpm for 7 h, the cells were collected and crude cell extracts were prepared using the bacterial soluble total protein preparation kit (GENMED Scientifics Inc., Arlington, MA, USA). The extracts were assayed for LDH activity using a kit to colorimetrically determine the total bacterial LDH activity (GENMED Scientifics Inc., Arlington, MA, USA) at pH 6.5 and at the same temperature as that of incubation (25°C ~ 50°C). One unit of the overall LDH activity was defined as the amount of enzyme required to transform 1 μmole NADH to NAD^+^ per minute. The protein concentration in the crude extracts was determined using the Bradford method, and bovine serum albumin was used as the standard. The LDH activity was divided by the corresponding protein concentration to calculate the specific LDH activity. The assays are performed in triplicates.

### Flask fermentation experiments

The flask fermentation experiments were carried out according to the previous publication [10,17] in 250 ml flasks with the working volume of 50 ml. Briefly, cells were grown in 50 ml of LB medium in a 250 ml flask at 37°C with shaking (200 rpm) for 8–10 h until cell density (OD_600_) of 2.0-2.5 was reached. Cells were collected by centrifugation and resuspended in a modified M9 medium and then inoculated into 50 ml of the modified M9 medium complemented with 5 g/l glucose in a 250 ml flask with the initial cell density (OD_600_) of 0.05. For cell growth experiments, the cultivation was carried out at shaking speed of 200 rpm and at different temperatures. For lactate fermentation, the cultivation was first carried out at 37°C and 200 rpm for 12 h, then 30 g/l glucose was added followed by stationary cultivation (anaerobic fermentation) for lactate formation at different temperatures. Calcium carbonate with a final concentration of 75 g/l was added for neutralization. Sampling was carried out during the cultivation. Modified M9 medium contained (per liter): 15.11 g Na_2_HPO_4_ · 12H_2_O, 3 g KH_2_PO_4_, 1 g NH_4_Cl, 0.5 g NaCl. One ml of filter-sterilized 1 M MgSO_4_, and 1 ml of filter-sterilized trace element solution containing (per liter) 2.4 g FeCl_3_ · 6H_2_O, 0.3 g CoCl_2_ · 6H_2_O, 0.15 g CuCl_2_ · 2H_2_O, 0.3 g ZnCl_2_, 0.3 g Na_2_MO_4_ · 2H_2_O, 0.075 g H_3_BO_3_, 0.495 g MnCl_2_ · 4H_2_O was added to a liter of the final medium.

### Fed-batch fermentation in bioreactor

A fed-batch fermentation experiment in bioreactor was carried out according to the method described [10,11,21]. A 25-l bioreactor (Bioflow110; New Brunswick Scientific Co., Inc., Edison, NJ), initially containing 11 l of the modified M9 medium as above in the “Flask fermentation experiments”, was used for L-lactate production from glucose. The two-phase fed-batch process was started by inoculating 600 ml fresh inoculum prepared by pre-culturing cells in LB medium as described above. The cells were cultivated in the aerobic condition followed by anaerobic fermentation. Anaerobic fermentation for L-lactate formation was initiated by ceasing air sparging and reducing agitation to 100 rpm when the cell density (OD_600_) reached about 30 (which is equal to about 11.4 g/l dry cell weight (DCW)). During the aerobic phase, glucose was supplemented with 30 g/l for cell growth and the culture was grown at pH 7.0 controlled by automatically feeding 25% (w/v) NH_4_OH solution and the dissolved oxygen tension controlled above 30% of saturation. During the anaerobic phase, the pH was controlled at 7.0 by the addition of 25% (w/v) Ca(OH)_2_. The residual glucose concentration was maintained above 10 g/l by adding glucose in four batches (649.5 g of glucose was added in total). The fermentations were stopped when the glucose was exhausted.

### Cultivation conditions for exploiting growth properties

Strains B0013-090B1, B0013-090B2 or B0013-090B3 heterologously expressing an L-lactate dehydrogenase (L-LDH) were examined for their growth rate and L-LDH activity. A shake flask fermentation test was carried out in a 250-ml flask with working volume of 50 ml at various temperatures from 25°C to 50°C for up to 14 h. The cell density was analyzed with the method as described below in the “Analytical methods”. The intracellular lactate dehydrogenase activities in strains B0013-090B1, B0013-090B2 and B0013-090B3 grown for 7 h were determined.

### Analytical methods

The cell mass was estimated by measuring the optical density at 600 nm (if CaCO_3_ or Ca(OH)_2_ had been added during fermentation, samples were pretreated with 1 M HCl at 20-fold of the sample volume to remove the suspended substances), and the dry weight of the cells was calculated using a standard curve (1*OD*_600_ = 0.38 g/l DCW) [10]. The glucose concentration was estimated using a glucose biosensor [10]. Samples were pretreated with H_2_SO_4_ (at 5% of the sample volume) to release organic acids that precipitated with CaCO_3_ or Ca(OH)_2_ during fermentation. Organic acid concentrations were measured by HPLC as described previously [10]. Organic acid and ethanol concentrations were measured by HPLC equipped with UV (210 nm) and refractive index detectors, using a Shodex SH-1011 column (Shodex SH-1011 H610009; Showa Denko K.K., Kawasaki, Japan) with 0.01 M H_2_SO_4_ as eluent (0.6 ml/min; 50°C). Lactic acid isomeric purity was measured by HPLC using a chiral column (CLC-L; Advanced Separation Technologies Inc., Whippany, NJ, USA), at room temperature, equilibrated with 1 ml/min of 5 mM CuSO4 as the mobile phase and detected at 254 nm with a UV detector.

## Results

### Metabolic engineering o*f E. coli* B0013 to produce L-lactate

**Figure 1:**
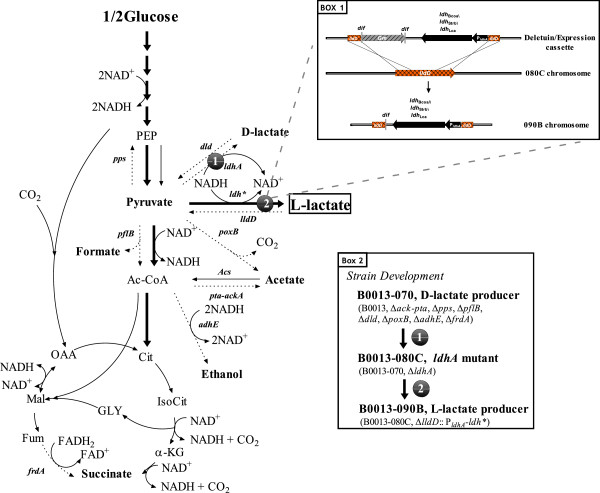
**The metabolic pathways for the production of L-lactate by engineered *E. coli *from glucose.** Relevant genes are shown. *pps*, PEP synthase; *pflB*, pyruvate formatelyase; *ldhA*, fermentative D-lactate dehydrogenase; *dld*, D-lactate dehydrogenase; *poxB*, pyruvate oxidase; *pta*, phosphotransacetylase; *ackA*, acetate kinase; *adhE*, alcohol dehydrogenase; *frdA*, fumaratereductase; *lldD*, FMN-dependent L-lactate dehydrogenase; *ldh*_Lca_, L-lactate dehydrogenase from *Lactobacillus casei* CICIM-CU B1192; *ldh*_Strb,_ L-lactate dehydrogenase from *Streptococcus bovis* CGMCC 1.1624; *ldh*_Bcoa,_ L-lactate dehydrogenase from *Bacillus coagulans* CICIM B1821. Box A presented the homologous recombination on the chromosome in B0013-090B series. The deletion/expression cassette was first electroporated into *E. coli* B0013-080C. The transformants were selected within LB medium containing gentamicin and the correct double replacement recombinants were confirmed by PCR. The second selection was carried out by incubating the correct recombinants in LB medium without gentamicin. The correct recombinants were confirmed by PCR. Box B outlined the construction procedure for B0013-090B1, and B0013-090B2 and B0013-090B3. P_*lacA*_-ldh* represented one expression cassette of *ldh*_Lca_, *ldh*_Strb_ and *ldh*_Bcoa_ under the control the promoter of *E. coli ldhA*. Abbreviations: PEP, phosphoenolpyruvate; Ac-CoA, acetyl-CoA; Cit, citrate; IsoCit, isocitrate; α-KG, α-ketoglutarate; Fum, fumarate; Mal, malate; OAA, oxaloacetate; GLY, glyoxylate.

For L-lactate synthesis in *E. coli*, the following genetic manipulations have been made: (1) construction of an L-lactate synthesis pathway by expressing a heterologous L-lactate dehydrogenase, (2) elimination of the D-lactate synthesis pathway by deleting *ldhA* encoding a D-lactate dehydrogenase, and (3) blocking of the L-lactate catabolic pathway by disrupting *lldD* encoding an FMN-dependent L-lactate dehydrogenase (Figure 1). First, a deletion cassette *ldhA*'-*dif*Gm-*ldhA*' for deletion of *ldhA* encoding D-lactate dehydrogenase was constructed and genetically transformed into *E. coli* B0013-070 according to the procedures described in the method section. A mutant, designated *E. coli* B0013-080C (B0013-070, *ldhA*::*dif*), was constructed and confirmed by PCR. This mutant failed to form D-lactate (Table 3). Then, the respective coding regions of *ldh*_Lca_, *ldh*_Strb_ or *ldh*_Bcoa_ encoding an L-lactate dehydrogenase from *L. casei, Str. bovis* or *B. coagulans* were cloned and their native promoters were replaced by the promoter of *E. coli ldhA*. This hybrid expression cassette (P_*ldhA*_-*ldh*_Lca_, P_*ldhA*_-*ldh*_Strb_ or P_*ldhA*_-*ldh*_Bcoa_) was then chromosomally integrated into the *lldD* locus in *E. coli* B0013-080C by electroporating the deletion/expression cassette *lldD*::P_*ldhA*_-*ldh*-*dif*Gm (here, *ldh* represents *ldh*_Lca_, *ldh*_Strb_ or *ldh*_Bcoa_) into B0013-080C followed by two cycles of selection in LB containing gentamicin and LB without gentamicin, yielding strain B0013-090B1(B0013, Δ*ack-pta*, Δ*pps*, Δ*pflB*, Δ*dld*, Δ*poxB*, Δ*adhE*, Δ*frdA,*Δ*ldhA,*Δ*lldD*::P_*ldhA*_-*ldh*_Lca_), strain B0013-090B2 (B0013, Δ*ack-pta*, Δ*pps*, Δ*pflB*, Δ*dld*, Δ*poxB*, Δ*adhE*, Δ*frdA,*Δ*ldhA,*Δ*lldD*::P_*ldhA*_-*ldh*_Strb_), or B0013-090B3(B0013, Δ*ack-pta*, Δ*pps*, Δ*pflB*, Δ*dld*, Δ*poxB*, Δ*adhE*, Δ*frdA,*Δ*ldhA,*Δ*lldD*::P_*ldhA*_-*ldh*_Bcoa_). Deletion of the *lldD* gene blocked the reflow pathway from L-lactate to pyruvate catalyzed by the FMN-dependent L-lactate dehydrogenase (Figure 1).

**Table 3:** Lactate formation in flask fermentation

**Strain**	**L-lactate (g/l)**^**a**^	**D-lactate (g/l)**^**a**^	**Yield of L-/D-lactate (%, w/w, based on total supplied glucose)**
B0013-090B1	20.16 ± 0.90	0.02 ± 0.00	58
B0013-090B2	16.19 ± 0.92	0.03 ± 0.00	46
B0013-090B3	25.59 ± 1.08	0.03 ± 0.00	73
B0013-070	0.00 ± 0.00	27.27 ± 1.06	78
B0013-080C	0.00 ± 0.00	0.01 ± 0.00	/

^a^The flask fermentation experiments were carried out in 250 ml flasks with the working volume of 50 ml. Briefly, cells were grown in 50 ml of LB medium in a 250 ml flask at 37°C with shaking (200 rpm) for 8–10 h until cell density (OD_600_) of 2.0-2.5 was reached. Cells were collected by centrifugation and resuspended in a modified M9 medium and then inoculated into 50 ml of the modified M9 medium complemented with 5 g/l glucose in a 250 ml flask with the initial cell density (OD_600_) of 0.05 in triplicates. The cultivation was first carried out at 37°C and 200 rpm for 12 h, then 30 g/l glucose was added followed by stationary cultivation (anaerobic fermentation) for lactate formation at different temperatures. Calcium carbonate with a final concentration of 75 g/l was added for neutralization. Sampling was carried out during the cultivation. Lactic acid concentration was measured by HPLC equipped with UV (210 nm) and refractive index detectors, using a Shodex SH-1011 column (Shodex SH-1011 H610009; Showa Denko K.K., Kawasaki, Japan) with 0.01 M H_2_SO_4_ as eluent (0.6 ml/min; 50°C). Lactic acid isomeric purity was measured by HPLC using a chiral column (CLC-L; Advanced Separation Technologies Inc., Whippany, NJ, USA), at room temperature, equilibrated with 1 ml/min of 5 mM CuSO4 as the mobile phase and detected at 254 nm with a UV detector.

A flask fermentation experiment was carried out at 37°C to evaluate the performances of the engineered strains. The results are summarized in Table 3. The total amounts of lactate produced by strain B0013-090B1, B0013-090B2 and B0013-090B3 was 20.16, 16.19 and 25.59 g/l from 35.0 g/l of glucose (5 g/l of glucose in LB medium and 30 g/l of glucose was added afterwards), respectively, while the total lactate produced by B0013-070 and B0013-080C was 27.27 g/l and 0.02 g/l respectively. The percentage of L-lactate in the total lactate produced by B0013-090B1, B0013-090B2 or B0013-090B3 was above 99.9%, while nearly no L-lactate was produced by B0013-070 and B0013-080C. These results indicated that all three L-lactate dehydrogenases were functionally expressed and capable of catalyzing L-lactate formation, and that D-lactate formation can be blocked thoroughly by deleting the *ldhA* gene. It is interesting to note that conversion efficiency of L-lactate and cell growth rate were obviously different among the recombinant *E. coli* strains carrying a different L-lactate dehydrogenase.

### Temperature as a factor for variation in cell growth and L-lactate synthesis

**Figure 2:**
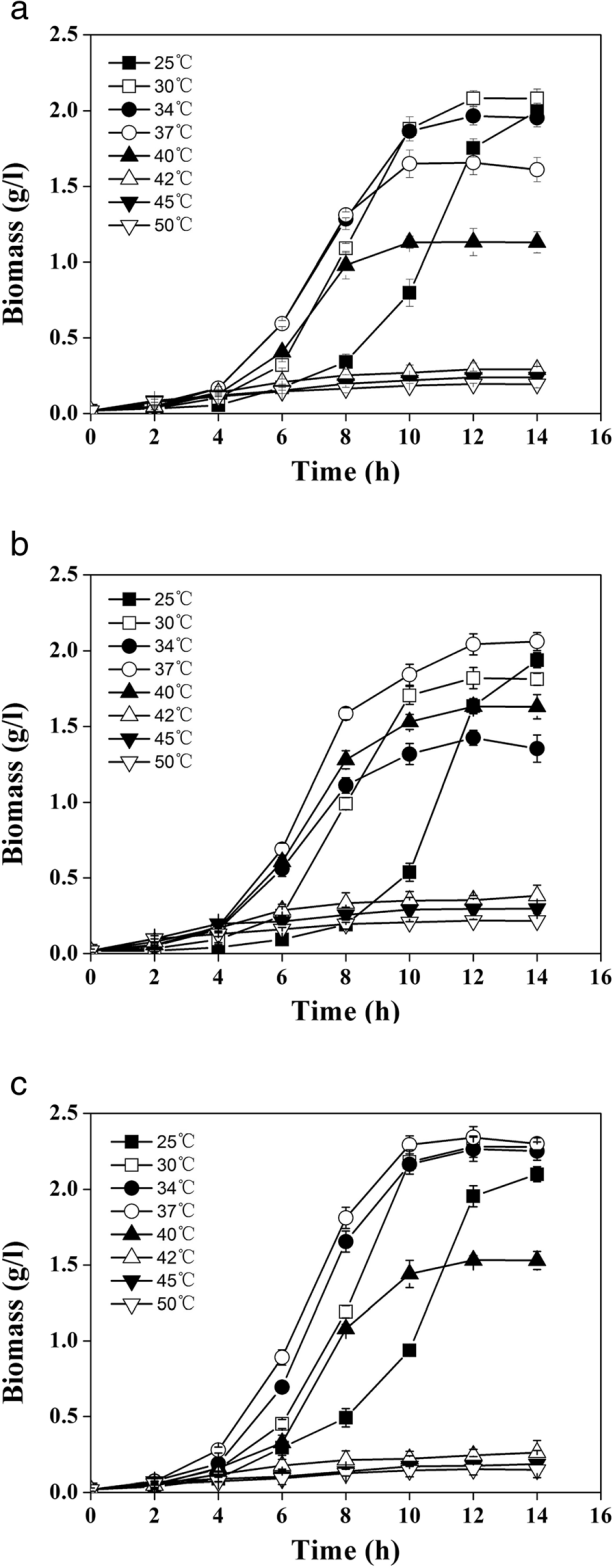
**The growth characteristics of *E*. *coli *strains 090B1 (a), 090B2 (b) and 090B3 (c) at various temperatures.** For the experiments, a shaking flask fermentation test was carried out in a 250-ml flask with working volume of 50 ml at various temperatures from 25°C, 30°C, 34°C, 37°C, 40°C, 42°C, 45°C to 50°C for up to 14 h. Sampling was carried out in every 2 hours. The cell density was colorimetrically analyzed at 600 nm. The experiments were carried out in triplicates.

**Figure 3:**
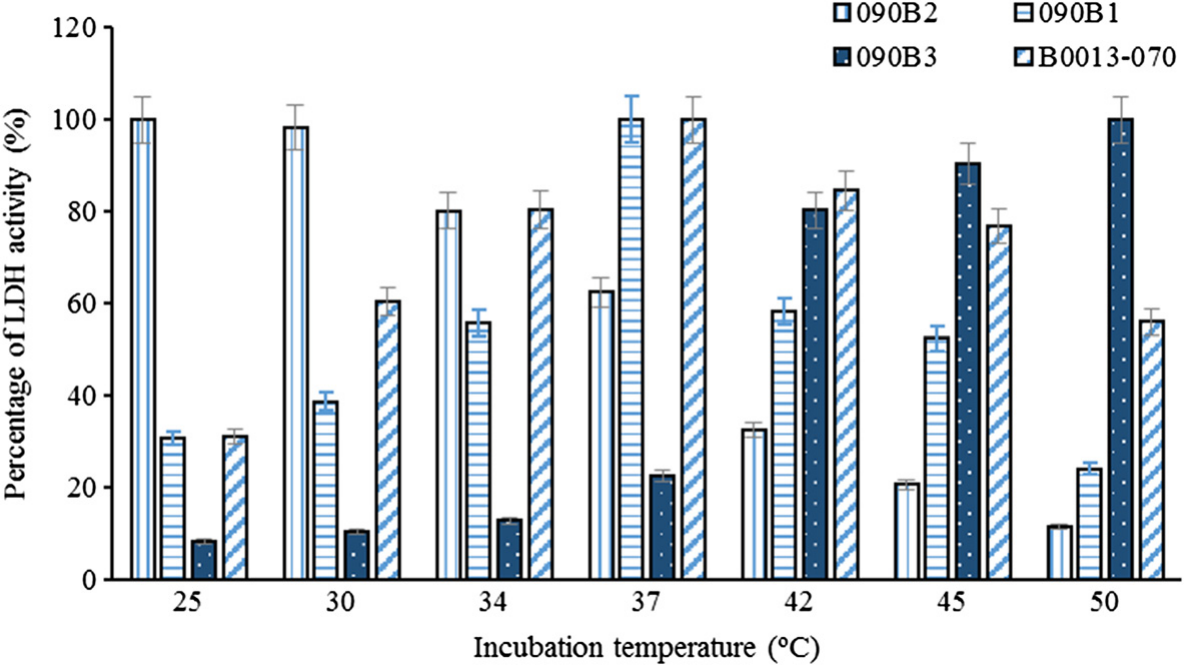
**Comparison of recombinant LDH activity expressed by strain 090B1, 090B2 and 090B3.** After growing while shaking at 200 rpm for 7 h, the cells were collected and crude cell extracts were prepared. The extracts were assayed for LDH activity at pH 6.5 and at the same temperature as that of incubation (among 25°C, 30°C, 34°C, 37°C, 42°C, 45°C or 50°C). The maximum specific LDH activity of each strain was standardized to 100%. The specific LDH activity of their parental B0013-070 was also presented as comparison. The experiments are performed in triplicates. Strain 090B1 showed maximum activity of LDH at 37°C (2.12 U/mg protein), Strain 090B2 yielded activity maximum at 25°C (1.95 U/mg protein), Strain 090B3 showed a activity maximum at 50°C (2.43 U/mg protein). In the parent strain, the highest activity (1.16 U/mg protein) was at 37°C.

Since cell growth and L-lactate formation share the same intermediate pyruvate derived from carbon catabolism in *E. coli*, it is expected that cell growth and L-lactate formation will compete for pyruvate and both processes would be affected by the incubation temperature. However, given the highly sensitive nature of the expressed L-lactate dehydrogenase to temperature, L-lactate formation is more susceptible than cell growth to fermentation temperature.Strains B0013-090B1, B0013-090B2 or B0013-090B3 heterologously expressing an L-LDH were examined for their growth rate. A flask fermentation test was carried out in a 250-ml flask with working volume of 50 ml at various temperatures from 25°C to 50°C for up to 14 h. The cell density was analyzed and the results are summarized in Figure 2. Maximum growth rate was achieved at temperature of 30 ~ 34°C for B0013-090B1 and 37°C for B0013-090B2 and B0013-090B3. When the cultivation temperature was higher than 42°C, obvious growth inhibition was observed for all three strains (Figure 2).Strains expressing different types of L-lactate dehydrogenases had different growth rates when incubated at different temperatures, suggesting that L-lactate dehydrogenase activity may be the key factor. To elucidate the intracellular L-lactate dehydrogenase activity patterns at different temperatures, the cells growing at different temperatures were collected and the intracellular activities determined. The results are summarized in Figure 3. The lactate dehydrogenase activity in B0013-090B3 was much less than that of B0013-090B1 or B0013-090B2 at both 34°C and 37°C. However, B0013-090B3 displayed about 3-fold higher lactate dehydrogenase activity at 42°C. These results indicate that incubation temperature can work as a sensitive factor to tune the activity of the expressed L-LDH and hence re-distributes the metabolic flux between the TCA cycle and pyruvate to L-lactate.

### L-Lactate production in a bioreactor using temperature as an adjustable parameter

**Figure 4:**
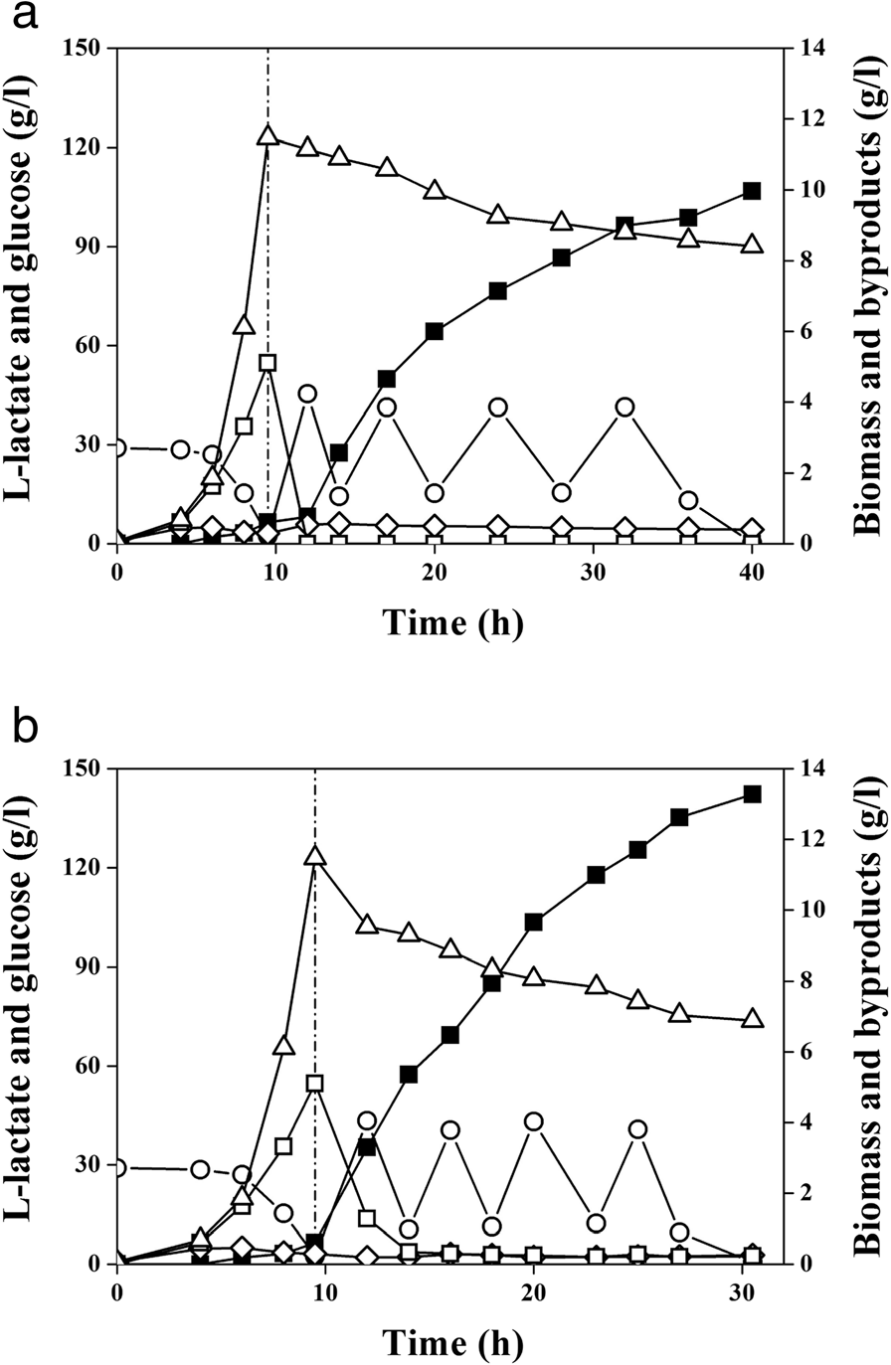
**Representative experiments (conducted in duplicate) showing production of organic compounds and dry cell mass during the aerobic and oxygen limited lactate fermentation by *E. coli *B0013-090B3 in a 25-l bioreactor.** The dotted line indicates the time when the culture was shifted from the aerobic cultivation to the oxygen limited production phase. Additions of glucose of 136.2, 136.2, 150.9 and 136.2 g were made to the bioreactor during the production phase. Open triangle: dry cell mass, closed square: lactate, open square: pyruvate, open diamond: acetate; open circle: glucose concentration. **(a)** The aerobic cultivation was carried out at 37°C with dissolved oxygen greater than 30% saturation by supplying 3–7 l/min air and 200–1000 rpm of agitation. The production phase was done at 37°C with 100 rpm of agitation and without aeration. **(b)** The aerobic cultivation was carried out at 37°C with dissolved oxygen higher than 30% saturation by supplying 3–7 l/min air and 200–1000 rpm of agitation. The production phase was done at 42°C with 100 rpm of agitation and without aeration.

As observed above, only *E. coli* B0013-090B3 harboring a *B. coagulans* L-lactate dehydrogenase gave higher LDH activity at ≥42°C and lower LDH activity at ≤37°C, which enabled us to consider temperature as a useful adjustable parameter for some metabolically-engineered strains. A temperature-shift fermentation process was developed and applied to check the growth and lactate formation efficiencies in a 25-liter bioreactor with final working volume of 20 liters. The fed-batch process was started by inoculating fresh inoculum of *E. coli* B0013-090B3 and cultivated in an aerobic condition at 37°C followed by anaerobic fermentation for L-lactate formation initiated by stopping aeration, reducing agitation and elevating temperature to 42°C when cell density (OD_600_) reached about 30. The results are summarized in Table 4 and Figure 4. Strain B0013-090B3 produced 142.2 g/l L-lactate with no more than 1.2 g/l of total by-products (mainly acetate, pyruvate and succinate). The average volumetric lactate productivity during the oxygen-limited fermentation phase and the lactate yield from glucose was 6.77 g/l h and 97% (g/g), respectively (Table 4). The overall volumetric lactate productivity and the oxygen-limited volumetric lactate productivity were improved up to 74.5% and 95.7%, respectively in comparison to those at 37°C (Table 4).

**Table 4:** Comparison of fermentation data (mean ± range of duplicate experiments) from the bioreactor experiments

**Strains**	**Overall volumetric lactate productivity (g/l h)**^**a**^	**Oxygen-limited volumetric lactate productivity (g/l h)**^**b**^	**Oxygen-limited specific lactate productivity (g/g h)**^**c**^	**Oxygen-limited specific glucose consumption rate (g/g h)**^**d**^	**Aerobic biomass yield (g/100 g glucose)**^**e**^	**Oxygen-limited lactate yield (g/100 g glucose)**^**f**^	**Lactate titer (g/l)**	**Yield (g/100 g glucose) (The molar yield)**^**g**^				
								**biomass**	**lactate**	**acetate**	**succinate**	**pyruvate**
**090B3**^**h**^	2.67 ± 0.10	3.46 ± 0.23	0.42 ± 0.00	0.50 ± 0.01	36.9 ± 0.2	83.0 ± 1.2	106.8 ± 1.1	5.8 ± 0.1	74.3 ± 1.2 (1.49 ± 0.02)	0.6 ± 0.1	0.3 ± 0.0	0.0 ± 0.0
**090B3**^**i**^	4.66 ± 0.12	6.77 ± 0. 30	0.98 ± 0.10	0.97 ± 0. 04	36.8 ± 0.1	97.0 ± 0.5	142.2 ± 0.7	4.9 ± 0.3	87.0 ± 2.1 (1.74 ± 0.04)	0.4 ± 0.1	0.1 ± 0.1	0.1 ± 0.2

^a^Average volumetric productivity for lactate during the overall fermentation process.

^b^Average volumetric productivity for lactate during the oxygen-limited phase.

^c^Average specific lactate productivity during the oxygen-limited phase. The molar yield was also presented in parentheses.

^d^Average specific glucose consumption rate during the oxygen-limited phase.

^e^Average biomass yield during aerobic phase.

^f^Average lactate yield during the oxygen-limited phase.

^g^Average yield during the overall fermentation process.

^h^The fermentation was carried out with aerobic cell growth at 37°C followed by fermentation at 37°C.

^i^The fermentation was carried out with aerobic cell growth at 37°C followed by fermentation at 42°C.

## Discussion

Metabolic engineering are focusing mainly on the manipulation of directly-related metabolic pathways, which is obtained by introduction and/or amplification of product-oriented pathways or by deletion or attenuation of competing pathways [22,23]. Currently, metabolic engineers tend to finely exploit a pathway or a reaction to make it compatible with the cell physiological properties [11,17,24-26].

Generally, cell growth depends on the synthesis of acetyl-CoA from pyruvate, which also acts as the precursor of lactate. Expression of a lactate dehydrogenase results in a certain percentage of pyruvate converted to lactate, thereby limiting metabolic flux for cell synthesis and eventually retarding cell growth. Specific to *E. coli*, intracellular activity level of the lactate dehydrogenase depends not only on the transcription and translation levels of its encoding gene, which is mainly affected by the unique cellular microenvironment in *E. coli,* but also on its original native biochemical properties as well. Due to these concerns, fine control of the transcription and translation of *ldhA* encoding NAD^+^-dependent D-lactate dehydrogenase and the intracellular activity level of the D-lactate dehydrogenase in D-lactate production was found to significantly improve both lactate yield and cell growth rate [11,17]. In that case, a novel strategy has been developed for finely-regulating lactate dehydrogenase expression in *E. coli*, in which a gene encoding a lactate dehydrogenase was under the control of a thermo-induced promoter [11,24]. In a bioreactor experiment using scaled-up conditions, strain B0013-070B produced 122.8 g/l D-lactate with an increased oxygen-limited productivity of 0.89 g/g h [11].

Most enzymes have a native temperature optimum but some have a broader plateau in activity for a certain temperature range. On the other hand, the enzymes belonging to the same super-family originating from different (micro-)organisms carry out the same reaction yet may show the different temperature optima. The effect of temperature on the activity of an enzyme are complex and can be considered as two forces acting simultaneously but in opposite directions. As the temperature is raised, the rate increases, but at the same time there exists a progressive inactivation (denaturation) of the enzyme protein. From the metabolic engineering’s point of view, temperature may become one of the most important factors that can be used to control the enzyme activity in a pathway and hence finely regulate the metabolic flux. In present case we used temperature to optimize the cell growth and L-lactate production.

In present studies, three L-lactate dehydrogenases with different temperature optima were functionally expressed in *E. coli*. The recombinant *E. coli* strain harboring an L-LDH from *B. coagulans* exhibited lower L-LDH activity at 37°C or below thus allowing robust cell growth at this temperature (Figures 2, 3). On the other hand, the enzyme showed higher activity at 42°C or above, which allowed cells to convert pyruvate to L-lactate efficiently, and the L-lactate titer reached 142.2 g/l (Figure 4, Table 4). To our knowledge this is the highest level of L-lactate production by recombinant *E. coli* cells ever reported.

The recombinant *E. coli* cells expressing an L-LDH from *L. casei* displayed a slow growth phenotype during the aerobic phase at 37°C, which can be attributed to the insufficient supply of substrate for cell growth due to channeling of metabolic flux by the effects of higher L-lactate dehydrogenase activity at the growth temperature (Figures 2, 3). Normal growth of the recombinant cells expressing the L-LDH from *B. coagulans* or S*tr. bovis* was observed at 37°C, consistent with the lower activity of L-lactate dehydrogenase at this temperature (Figures 2, 3). These observations strongly suggest that it would be essential to either investigate the enzymatic properties or use a combination of promoter, temperature or pH conditions of a specific target enzyme before engineering a metabolic pathway. Different requirements for cell growth and target product formation conferred by a target enzyme can be utilized to rationally design and construct efficient metabolic pathways. Additionally, the desired enzymatic properties of a specific enzyme referred to a specific pathway or a reaction can be obtained either by cloning of genes obtained from microorganisms of extensive bio-diversity [27,28] and/or by gene manipulation in the laboratory using modern molecular tools [29,30].

In conclusion, the rapid growth characteristics and clearly defined metabolic pathway information in *E. coli* make it one of the most excellent lactate producers. The scale-up production of lactate by *E. coli* was limited by the competition relationship between cell growth and lactate synthesis in which lactate dehydrogenase activity is a critical factor. Furthermore, as described in present studies, properties, especially the thermodynamics of an enzyme can be effectively used as a powerful alternative tool to finely regulate a metabolic pathway and its metabolic flux by controlling temperature during cultivation/fermentation. This approach is convenient and economical to operate in any biotechnological process.

## Competing interests

The authors declare that they have no competing interests.

## Authors’ contributions

DN conceived the study, participated in its design and carried out the molecular genetic studies. KT carried out the molecular manipulation and the fermentation experiments. DN, KT, BAP, MW, ZW, FL and SS analyzed the data and prepared the manuscript. All authors read and approved the final manuscript.
